# Consumer Understanding of the Date of Minimum Durability of Food in Association with Quality Evaluation of Food Products After Expiration

**DOI:** 10.3390/ijerph17051632

**Published:** 2020-03-03

**Authors:** Dorota Zielińska, Beata Bilska, Katarzyna Marciniak-Łukasiak, Anna Łepecka, Monika Trząskowska, Katarzyna Neffe-Skocińska, Marzena Tomaszewska, Aleksandra Szydłowska, Danuta Kołożyn-Krajewska

**Affiliations:** 1 Department of Food Gastronomy and Food Hygiene, Institute of Human Nutrition Sciences, Warsaw University of Life Sciences—SGGW, Nowoursynowska 159C St., 02-776 Warsaw, Poland; dorota_zielinska@sggw.pl (D.Z.); monika_trzaskowska@sggw.pl (M.T.); katarzyna_neffe_skocińska@sggw.pl (K.N.-S.); marzena_tomaszewska@sggw.pl (M.T.); aleksandra_szydlowska@sggw.pl (A.S.); danuta_kolozyn_krajewska@sggw.pl (D.K.-K.); 2 Department of Food Technology and Assessment, Division of Fat and Oils and Food Concentrates Technology, Institute of Food Sciences, Warsaw University of Life Sciences—SGGW, Nowoursynowska 159c, 02-776 Warsaw, Poland; katarzyna_marciniak_lukasiak@sggw.pl

**Keywords:** date labels, food quality, food safety, food waste

## Abstract

Food labelled with a “best before” date has a long shelf life. This study aimed to examine the respondents’ knowledge and understanding regarding the labelling on food products, as well as to assess the microbiological, physico–chemical and the sensory quality of selected durable food products on and after the date specified by the manufacturer. Two methods were used—a survey and laboratory tests. It was found that the majority of respondents have difficulty distinguishing and understanding the terms on the label and that a significant proportion of the respondents consume food products after the “best before” date. Laboratory tests of milk, pasta, mayonnaise and jam confirmed the microbiological safety of the products even six months after the “best before” date. Other features (texture, colour and sensory quality) slightly changed after one month for milk and mayonnaise (the colour had become more yellow) and after three months for pasta (its hardness had decreased) and jam (it had become browner). The possibility of extending the “best before” dates of selected durable foods could be considered, which could allow such products to legally be handed over to public benefit organisations, thereby reducing food wastage.

## 1. Introduction

The Codex Alimentarius [1] recommends introducing two types of dates on food products—one relating to food health safety and the other to quality. According to European Union (EU) Regulation [2], the “date of minimum durability of a food” defines the date until which the food retains its specific properties when properly stored.” However, in the case of foodstuffs which spoil quickly from a microbiological point of view and may therefore pose a direct threat to human health after a short time, the “best before” date is replaced by the “use by” date. Under Polish law [3] foods labelled with a “best before” or “use by” date may be marketed until that date.

Different wording on date labels and misunderstandings of the meaning of expiry dates directly lead to throwing away food more frequently [4,5,6,7,8,9]. Research suggests that consumers waste food products which are approaching the date of minimum durability for health and safety reasons [6,10], as well as for reasons related to food quality [11,12]. A study by Wilson et al. [8] showed that the dates on labels had an impact on consumer behaviour and, consequently, on food waste. These results are consistent with other studies which indicate that consumers have a worse view of product quality approaching the expiration date [6,7,9,11].

Easy access to food and reliance on the date of minimum durability printed on the label may prompt consumers to dispose of food after it has expired [13]. On the other hand, it should be taken into account that some consumers do not check the dates on the packaging or consciously consume food after the “best before” date. At the same time, there is a lack of objective data on the quality of products and their safety beyond the expiration date. Considering all of the above, as well as the current efforts to reduce food waste, an attempt should be made to provide complete and reliable data that could be used to construct new food policy regulations in the EU and in the world. The new research approach should account for, on the one hand, the attitudes and behaviour of consumers in this respect and on the other hand the quality of food products after the minimum durability date recommended by the manufacturer has passed.

The aim of this study was to learn about the respondents’ knowledge and understanding regarding the labelling on “best before” and “use by” food labels, as well as to assess the possibility of prolonging the date of minimum durability based on microbiological, physico–chemical and sensory quality tests of selected durable food products on and after the date of minimum durability provided by the manufacturer.

## 2. Materials and Methods

**Figure 1:**
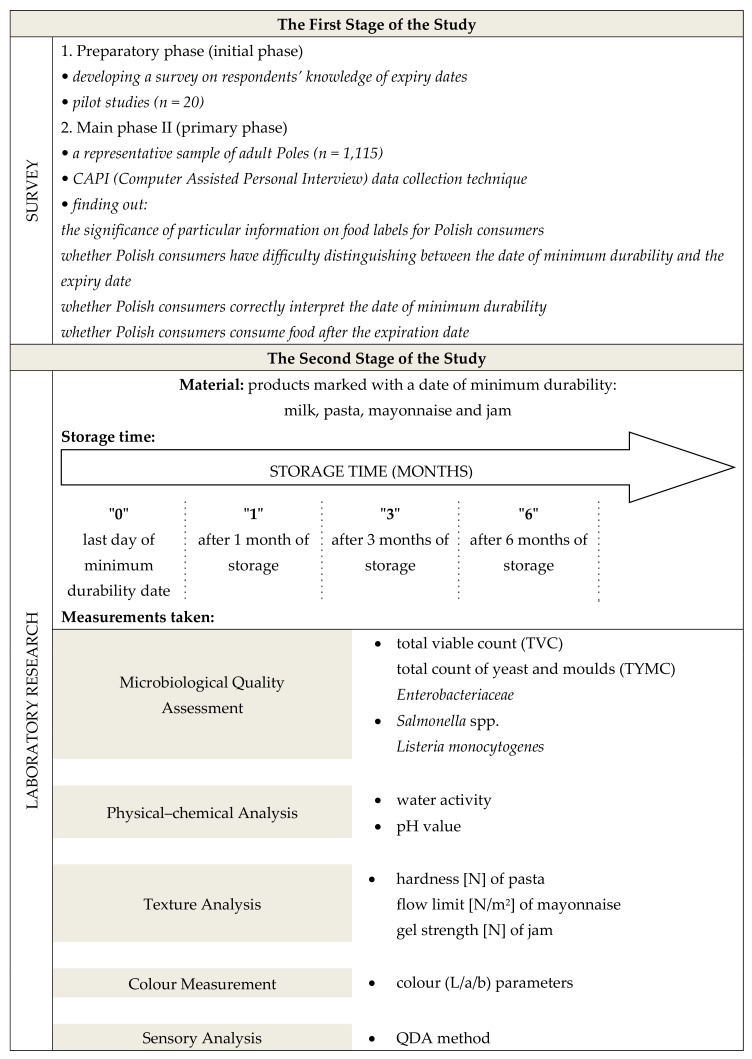
Study outline.

We used two methods—a survey conducted among respondents and laboratory tests of food products. The first stage of the study (survey) aimed to examined consumer knowledge and understanding of food labelling, related to the expiration date. As a logical consequence, after results evaluation, we decided to conduct the second stage of the study (laboratory tests) concerning four selected, typical, often used by Polish consumer products. The products were evaluated with a microbiological safety study, as well as physico-chemical and sensory analysis during storage after “best before” date expired (Figure 1).

### 2.1. Survey Study

#### 2.1.1. Sample Collection

The study sample amounted to 1115 people and was representative of all Poles over 18 years of age. The stratification took into account their place of residence and demographic characteristics broken down into 16 voivodships, 7 size categories of cities, gender and age (5 groups). The actual study was preceded by a pilot study on a sample of 20 people. The selection of the sample ensured that the study is representative and the structural division of the sample according to gender, age and place of residence does not differ significantly from the entire population. In the study, we used the CAPI technique (Computer Assisted Personal Interview), which is a method of collecting data that involves interviewing a respondent using mobile devices. The data were collected in February and March 2019. The maximum estimation error for a random sample of 1,000 people is +/- 3.1%. The sociodemographic data are presented in Table 1.

#### 2.1.2. Survey

The study was conducted using a specially designed questionnaire, which consisted of two sections. The first section contained questions about what consumers do with unopened food. This section of the survey contained closed questions with a choice of one of six answers about eating food after the expiration date. In the next question, respondents were asked to choose the products labelled “best before” from six food products. When asked about the importance of seven pieces of information on the label, a 5-grade scale was used, from “definitely agree” to “definitely disagree.” Another question asked was whether the information on the packaging marked as “use by” and “best before” mean the same thing (possible answers: “yes,” “no” or “it’s hard to say”). The respondents were asked to choose one of five answers describing the phrase “best before.” The second section contained questions regarding the sociodemographic description of the respondents (Table 1).

### 2.2. Quality of Food Products

#### 2.2.1. Food Products Characteristic

The research material was food products purchased on the Polish market, made available for retail sale. The products differed in methods of processing and origin of ingredients and represent typical, often consumed foodstuff in Poland. The samples were tested on the last day according to the minimum durability date and 1, 3 and 6 months after this date. Until the analyses were carried out, the products were stored in their factory-sealed packages, in a laboratory room, free of foreign odours and at room temperature. All analyses in this study were performed in three replicates. The details of the research material are shown in Table 2.

Following the applicable European legal requirements in the area of hygiene and safety of food production, all manufacturers declared the production of the above-mentioned products to be in accordance with the requirements of good hygienic practices (GHP) and good manufacturing practices (GMP), as well as with the guidelines of the system for ensuring food health safety, Hazard Analysis and Critical Control Points (HACCP) [14].

#### 2.2.2. Microbiological Quality Assessment

The total viable count (TVC) according to Reference [15], the number of bacteria from the *Enterobacteriaceae* family [16] and the number of yeast and mould (TYMC) [17], on the “best before” date and after 1, 3 and 6 months of storage were measured. In addition, the material was tested for the presence of *Salmonella* according to Reference [18] and *Listeria monocytogenes* according to Reference [19] on the “best before” date and after 6 months of storage.

#### 2.2.3. Physico–Chemical Analysis

The measurements of water activity were carried out in accordance with Reference [20], using an AQUALAB Pawkit Water Activity Meter (METER Group, Inc., Washington, USA). The samples were kept at 25°C for 1 h to obtain the right temperature.

The pH measurement of the milk, jam and mayonnaise was performed using an F 20 pehameter (Mettler Toledo) with a precision of up to 0.01, according to the device’s manual. The pH measurement of each sample was performed in triplicate.

The flow limit measurement [N/m^2^] of the mayonnaise was done using a Brookfield DV3T rotational viscometer (Brookfield Eng. Lab., Inc., USA). The following parameters were used—test speed–5 rpm and measurement temperature–20±1°C. The analysis was carried out using a V-73 spindle at a test speed of 5 rpm. The measurement was performed in triplicate and the arithmetic mean was taken as the result.

The hardness of the pasta samples was analysed using a TA.XT Plus Analyzer (Stable Micro Systems, UK). Hardness was determined using a P/36R head at a test speed of 2 mm*sec^−1^. The measurement was performed in five iterations and the arithmetic mean was taken as the result.

The gel strength mechanical properties tests were carried out on the jam using a TA-XT2i texturemeter (Stable Micro Systems, UK). Hardness was determined using a P/0.5R head. The compression speed was 2 mm*sec^−1^. The measurement was performed in five iterations and the arithmetic mean was taken as the result.

The colour of the products was characterised using the L, a b system proposed by the International Commission on Illumination (CIE) in the work of Papadakis et al. [21]. L refers to the luminosity or lightness component, a to the intensity of red (+) and green (−) and b indicates the intensity of yellow (+) and blue (−); these constitute the chromaticity coordinates. All samples were analysed in terms of these parameters using a Minolta CR-310 colorimeter (Konica-Minolta, Osaka, Japan) that was previously calibrated with a white standard tile. Five repetitions were made of each measurement.

#### 2.2.4. Sensory Analysis 

The sensory analysis was done using the Quantitative Descriptive Analysis (QDA) method according to Reference [22]. The analyses were conducted on the date of minimum durability and 1 month, 3 months and 6 months after the date of minimum durability. The descriptors were chosen and defined during a panel discussion and then verified in a preliminary session. The sensory attributes were measured in order to quantify the quality of the products. An unstructured, graphical scale of 10 cm was afterwards converted to numerical values (0–10; conventional units, c.u.). The assessments were carried out with the participation of a team of 16–20 employees of the Department of Food Gastronomy and Food Hygiene Warsaw University of Life Sciences – SGGW. The members of the evaluation team were trained in the appropriate methodology of analyses carried out and tested for sensory sensitivity.

### 2.3. Statistical Analysis 

#### 2.3.1. Statistical Analysis of Quantitative Research

We performed an analysis of the variation in the survey answers based on a quantitative scale depending on metric features:

- Mann–Whitney U test for variation of the results according to gender
- Kruskal–Wallis test for variation of the results depending on age, education, subjective assessment of financial situation and place of residence.

In order to answer the question of whether the metric features differentiate the survey answers based on a qualitative, dichotomous scale, we made calculations using the chi-squared independence test. The chi-squared independence test was used to verify the relationship between the two independent variables.

#### 2.3.2. Statistical Analysis of Food Quality Data

Analysis of variance and Student’s t-test were used to study the data from microbiological, physical–chemical and instrumental (texture and colour analysis) tests. Statistical significance was recognised when *p*< 0.05. Data from the sensory tests were analysed with the ANOVA and Tukey test at *p*< 0.01. All tests were carried out using Statistica software version 12.1 PL (StatSoft, Krakow, Poland).

## 3. Results

### 3.1. Survey Results

Almost half of the respondents reported that they do not consume food after the expiry date and one in five respondents admitted that he or she consumes expired durable products, such as cereal, pasta and so forth (Table 3). Only 7% of the respondents said that they consume food after the expiration date, regardless of its type. The behaviour of Polish consumers in relation to expired products was conditioned by such sociodemographic features as gender, education, place of residence and their subjective assessment of their financial situation (Table 3). Women more often than men reported consuming expired durable products and a reluctance to consume products after the expiration date. Men, on the other hand, more often indicated that they had difficulty responding to the question because they do not check the expiration dates. People with a higher education, living in large cities and assessing their financial situation as good more often denied consuming expired products than people with lower education, living in the countryside or a small city and assessing their financial situation as poor (Table 3).

The significance of particular information contained on the label when purchasing food products was determined (Table 4). The expiry date turned out to be important for the vast majority of respondents. Consumers with a higher education are more likely (96.3%) to pay attention to information related to the expiry date than are respondents with a secondary-school (91.9%), basic vocational (90.0%) or primary-school (91.4%) education. Similar relationships were also observed in the case of other information placed on labels, that is, consumers with a higher education attach greater importance to the nutritional and caloric values, the list of ingredients or the storage conditions recommended by the manufacturer compared to consumers with other levels of education. When considering the respondents’ place of residence, it was noted that residents of cities with a population of 200,001–500,000 attach the most importance to the expiry date on the product label (98.7%), while the inhabitants of smaller cities, that is, 100,001–200,000 residents (81.0%), found it less important.

Almost half of the respondents do not see a difference between the phrases “use by” and “best before,” and one in five respondents says that they have difficulty commenting on the issue (Table 4). The respondents’ place of residence turned out to be the only variable that significantly differentiates the percentage of answers. Inhabitants of large cities had the most difficulty indicating the correct answer. Only 28.2% of such inhabitants gave the correct answer. Rural residents (34.8% of correct answers), inhabitants of cities with up to 50,000 inhabitants (38.0% of correct answers), cities of 50,001–100,000 inhabitants (49.7%), cities of 100,001–200,000 inhabitants (32.6%) and cities with 200,001–500,000 inhabitants (37.1%) all dealt with the question much better.

Most respondents indicated that the date of minimum durability “best before” means the date after which the product becomes unsafe for the consumer (Table 3). A slightly smaller percentage of Poles gave correct answers, indicating that this is the date after which the food product loses its quality and the cannot be sold. Women knew that exceeding the date of minimum durability could mean a loss of product quality far more often than men did (41.4% vs. 33.2%).

### 3.2. Results of Food Quality Testing

#### 3.2.1. Microbiological Quality Assessment

The microbiological quality of selected durable food products is presented in Table 5.

For all samples of the food tested, the total number of aerobic, mesophilic (TVC) microorganisms was low, near the detection limit. The same was true for the number of yeast and mould cells (TYMC) and a count of bacteria from the *Enterobacteriaceae* family. In the case of jam, the number of microorganisms did not exceed 3.0 log CFU/g; in the case of milk and pasta, the highest value was 2.5 log CFU/g and 2.0 log CFU/g in the case of mayonnaise. No increase in the number of microorganisms was observed in any of the examined food groups, which proves stable microbiological quality. None of the tested durable food samples were found to contain *Salmonella* spp. and *Listeriamonocytogenes* on the minimum durability date, nor after 6 months of storage.

#### 3.2.2. Physico–Chemical Analysis

Table 6 presents values of water activity and pH levels of food products tested during storage.

The water activity of the milk, mayonnaise and jam was close to 1.00, whereas the water activity of the pasta was approx. 0.5. The water activity of all products on the date of minimum durability, as well as after 1, 3 and 6 months, was almost constant and the changes were insignificant and rather small.

Similarly, it was observed that the pH value of the food products did not change significantly during the six months of storage. The mayonnaise had a pH value in the range of 3.79–3.80, the jam had a pH in the range of 3.64–3.65 and the pH value of the milk, tested throughout the 6-month storage period, was in the range of 6.56–6.61.

In the case of mayonnaise, the flow limit (the lowest stress value at which the sample begins to deform) was analysed as a parameter of texture; no significant changes were noted during storage. Similarly, jam had unchanging gel strength parameters. However, when measuring the hardness of the pasta, a significant reduction was observed after 1 month of storage. This state was maintained for up to 6 months of storage (Table 7).

Table 8 demonstrates the changes in the parameters for colour of the food products tested during storage.

For milk and mayonnaise, there were observed significant changes in the values of the parameters L (brightness) and a* (representing the proportion of green—values below zero—or red—values above zero), which became visible after 1 month of storage after the expiry date and persisted until the end of the storage period. The colour of the pasta changed significantly after 3 months of storage beginning from the date of minimum durability, in terms of the values of parameters a* and b*. In the case of jam, no change in colour was observed.

#### 3.2.3. Sensory Analysis

**Figure 2:**
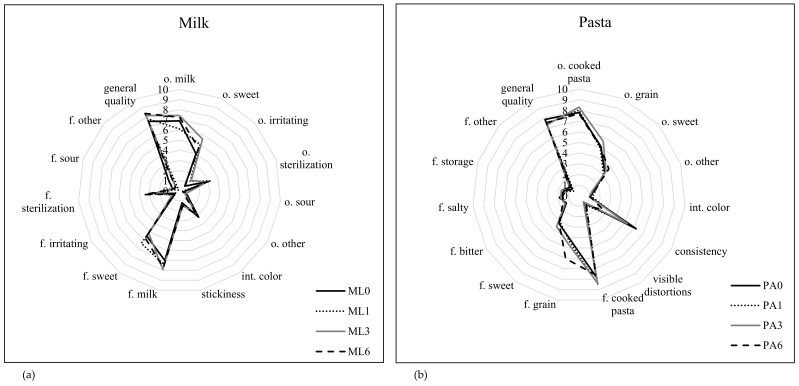
Sensory evaluation of the milk (**a**) and pasta (**b**).Explanatory notes: o–odour; int–intensity; f–flavour; ML–milk, PA–pasta; ML0 and PA0–time 0 (“best before” date); ML1 and PA1–1 month after the “best before” date; ML3 and PA3–3 months after the “best before” date; ML6 and PA6–6 months after the “best before” date.

**Figure 3:**
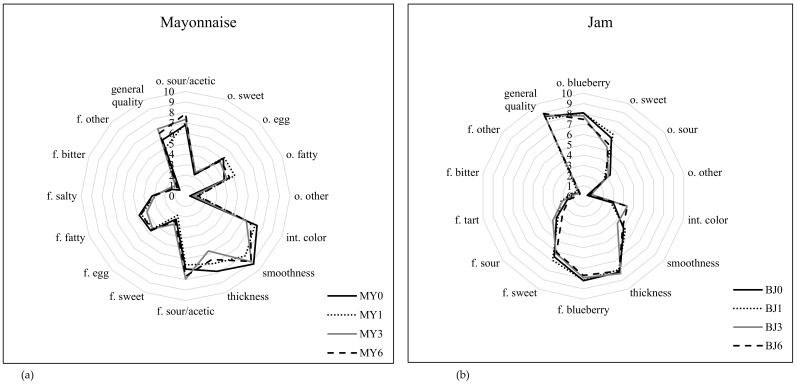
Sensory evaluation of the mayonnaise (**a**) and blueberry jam (**b**).Explanatory notes: o–odour; int–intensity; f–flavour; MY–mayonnaise, BJ–blueberry jam; MY0 and BJ0–time 0 (“best before” date); MY1 and BJ1–1 month after the “best before” date; MY3 and BJ3–3 months after the “best before” date; MY6 and BJ6–6 months after the “best before” date.

The tested food products were characterised by different levels of sensory quality (Figure 2 and Figure 3). Milk on the date of minimum durability was of good sensory quality and did not significantly change during storage. The main flavours indicated by consumers were a milky odour (6.9–7.4 c.u.), a sweet odour (4.0–5.6 c.u.) and a sterilisation odour (3.0–2.8 c.u.). The colour of the milk was white and the viscosity was low. The flavours were dominated by a milky flavour (7.0–7.9 c.u.) and a sweet flavour (5.2–6.4 c.u.). The overall quality of milk was high (7.5–8.4 c.u.). When tested 1, 3 and 6 months after the date of minimum durability, the colour of the milk changed slightly towards a more yellow colour and the viscosity of the milk decreased; however, the changes were not statistically significant (*p*> 0.01).

On the “best before” date, the pasta was characterised by very good sensory quality. The odour was dominated by an odour of cooked pasta (7.7–8.3 c.u.) and a grain odour (4.9–5.6 c.u.). The colour of the pasta was assessed as intense yellow, with a semi-hard consistency and no visible distortions of the form. The most intense flavours indicated by consumers were a flavour of cooked pasta (7.6–8.5 c.u.) and a grain flavour. The overall quality of the pasta on the date of minimum durability was 7.4–7.8 c.u. One month after the “best before” date, a decrease in the consistency of the cooked pasta was observed (*p*< 0.01). An increase in the intensity of the grain flavour (by 1.3 c.u.) was also noted after 6 months of storage.

The mayonnaise was characterised by a sour odour (6.8–7.9 c.u.), an egg odour (4.7–5.2 c.u.) and a fatty odour (4.0–5.1 c.u.). The colour of the mayonnaise was slightly yellow; it was assessed as smooth and thick. The mayonnaise flavours were dominated by an intense sour flavour (6.6–7.9 c.u.), an egg flavour (4.4–4.7 c.u.) and a fatty flavour (4.0–4.8 c.u.). The overall quality of the mayonnaise was average (5.0–6.9 c.u.). When tested 1, 3 and 6 months after the “best before” date, a significantly more intense sour odour was observed. The colour of the mayonnaise did not change, though the smoothness and thickness decreased (*p*< 0.01).

The blueberry jam had an intense blueberry odour (7.5–8.1 c.u.) and an intense sweet odour (5.6–6.7 c.u.). The colour of the jam was assessed as intense purple, which changed slightly towards brown after 3 months of storage (*p*< 0.01). The consistency of the jam was not smooth but the thickness of the jam was found. The flavours were dominated by an intense blueberry flavour (7.7–8.2 c.u.) and a sweet flavour (6.0–6.9 c.u.). The overall quality of the blueberry jam was very high (8.3–8.9 c.u.). One month after the “best before” date, no major differences in the odour, texture, flavour or overall quality of the blueberry jam was detected. Three months after the “best before” date, however, a decrease in the intensity of the blueberry odour and sweet odour was noted (*p*> 0.01). Despite the changes in the flavour of the jam, the overall quality of the jam still remained high after 6 months of storage.

## 4. Discussion

The results of this study revealed that almost half of the Polish respondents, regardless of their age, said that they never consume food after the expiry date. Neff et al. [5] reported that consumers over 34 years of age discarded all of the tested food products based on the expiry date less often than younger people. Other studies also indicate that age can have a significant impact on food management [23].

Our study showed that two pieces of information on the packaging had the greatest importance for most respondents—the price and the expiry date, which was also stated by Grunert et al. [24]. In contrast, Angowski et al. [25] found that the “best before” date was third among the factors that had a significant impact on consumers’ selection of dairy products. As our results suggest, women check expiration dates more often than men. This was also noted by Neff et al. [5] and the European Commission Report [23]. We found that women eat food after the expiration date less often than men do. Research Neff et al. [5] did not confirm this observation.

As many as 42% of the Polish respondents believe that the expressions “use by” and “best before” signify the same thing and one in five chose the answer, “It’s hard to say.” Almost 40% of the Polish respondents mistakenly believe that the term “best before” refers to food health safety, irrespective of sociodemographic variables. In the study by Neff et al. [5], a similar percentage of respondents mistakenly believed that the term “use by” is related to product quality. Research carried out by Kosa et al. [26] showed that 31% of respondents correctly identified the date of minimum durability and 18% correctly specified the definition of the term “use by.” The European Commission Report [23] points to a greater understanding of the date of minimum durability among respondents from 28 EU countries (47% of correct answers) and the impact of age and education. A larger percentage of respondents aged 25–39 and people who completed education at a later age knew the meaning of the terms “use by” and “best before” than people from other age categories and with less education [23]. Almost 38% of Polish respondents correctly matched the answer to the term “use before.” The results of our study are similar to those obtained by WRAP [9] (39% of correct answers). In contrast, 10% of American respondents believed that consumption of the product after the date of minimum durability comes with a health risk [27]. Also, the results of a study by Hall-Phillips and Shah [28] confirm that understanding expiration dates is a major challenge for consumers, who sometimes make the wrong decisions that lead to food waste. Koivupuro et al. [29] named misreading labels as one of the factors that lead to the disposal of food.

Our research shows that most respondents have difficulty distinguishing between the terms on the label and (consciously or not) consume products after the “best before” date. This finding became the starting point for the next stage of research, which prompted us to assess the safety and quality of selected food products from the “durable products” group. The results of our analysis indicate that the microbiological quality of four food products was at a satisfactory level throughout the study period (6 months from the “best before” date). In the case of milk, the manufacturer declared using ISO 22000 systems during production. Similarly, the mayonnaise producer claimed to have product quality certificates. These factors can positively affect product safety.

The method of packaging also affects the maintenance of high and consistent food quality [30]. The producer of mayonnaise asserted that the packaging was done in a protected environment, while the milk producer claimed to use aseptic packaging.

Other physico–chemical parameters of food, such as water activity or pH value, may affect food durability, thus inhibiting the growth of microorganisms. Free water in the product can take part in biochemical reactions or used by microorganisms. Thus, the higher the a_w_ is, the more easily the product can be contaminated [31]. The minimum value of water activity necessary for bacterial growth is 0.90; for most yeasts, a_w_ = 0.80 and for most moulds, a_w_ = 0.70. It is widely accepted that microbes cannot grow in foods with an aw of <0.60 [32]. In our study, only pasta had a low water activity value (<0.6), which indicates its long shelf life. Therefore, the pH of this product was not tested. In the case of other products with high water activity (a_w_> 0.60), thermal methods of food preservation should be used, what can definitely increase their durability. In our products, the milk was ultra-high-temperature processed and the jam was pasteurised.

The low pH value may limit the growth of saprophytic microflora. In the products tested, the pH value of the jam was about 3.6, while the pH value of the mayonnaise was about 3.8. These are low values that can guarantee stable microbiological quality, especially with respect to bacteria. Most mesophilic, aerobic microorganisms tolerate pH in the range of 4.5–9.0—only yeasts and moulds can grow in an environment with a pH of 2.0–8.8 [33]. Similar results for pH value were obtained by Ghazaei et al. [34] when examining low-fat mayonnaises. The Polish Standard [35] only contains requirements for general acidity. The total acidity of mayonnaise may not be greater than 0.8% (m/m), calculated as acetic acid and of mayonnaise with flavour additives, no greater than 0.9% (m/m), calculated as acetic acid—regardless of whether they are high- or low-fat products. In turn, the pH of jams is an important factor that provides optimal conditions for viscosity formation [36]. The determined pH was suitable for gelling, which translates into product durability during storage. The pH for milk is the basic criterion for testing the quality of technological suitability. The pH value of the milk during the 6-month storage period was in the range of 6.56–6.61, which indicates good product quality because fresh milk normally has a slightly acidic pH—between 6.5 and 6.8.

When considering stability, the microbial and chemical safety aspects must be considered first before sensory properties. The microbial stability and safety of most foods is based on a combination of several preservation factors, called hurdles. In achieving the desired safety by only one hurdle, rigorous processing standards need to be applied. This causes significant damage to the nutritional and sensory quality of foods. For this reason, it is important to have a multi-hurdle approach for developing safe and wholesome food products [37,38]. In the case of the products tested in this study, at least two methods were used to make them more durable, which allowed for the food safety to be maintained.

Adequate sensory properties are crucial for consumers when choosing food. Appearance, taste, smell and even the noises made while eating can cause food to be accepted or rejected [39,40]. A trained team of experts took part in the sensory analysis of this study. It is worth noting that some parameters are not always visible to the naked eye, which is apparent in the study of colour and texture. Texture and colour are key determinants of food quality. Texture analysis is performed mainly to determine the impact of technological processes on the raw materials’ properties and on product quality. Combined with the colour, it also affects the appearance of the product and consequently consumer acceptance [41].

Hardness is also an important determinant of the usefulness and culinary quality of pasta [42]. When it comes to this product, a gradual decrease in hardness was observed both in the hardness assessment of the texture tests and in the sensory analysis. Greater softness of the pasta was observed 6 months after the date of minimum durability than on the expiry date. In the case of the pasta colour analysis, the L value was in the range of 75.72–76.40, which points to a high degree of brightness. According to Rosa et al. [43], even if some consumers could accept dark pastas, lighter-coloured pastas are more frequently acceptable because consumers are accustomed to eating wheat pastas.

In the case of mayonnaise, after 3 months of storage, the researchers noticed changes in its consistency, which was reflected in lower yield stress values, although the statistical analysis did not show significant differences in this parameter over time. The mayonnaise and milk colour changes coincided in the texture analysis and the sensory analysis. The milk and mayonnaise had become lighter but more yellow over time. The colour of milk is perceived by consumers to be indicative of its purity and richness. Cow’s milk contains the pigments carotene and xanthophyll, which tend to give a golden yellow colour to the milkfat [44]. Similar findings were obtained by Daszkiewicz and Rymkiewicz [45], who studied changes in UHT milk colour during storage. They found that UHT milk stored at 20°C was characterised by greater changes in colour, with an increase of red and yellow and in the yellowness index. Storage changes of mayonnaise’s colour to yellower were also found by Lennersten and Lingnert [46].

Slightly different results of colour measurement in the texture and sensory analysis were obtained for jam. Consumers reported a browning of the jam 3 months after the date of minimum durability, which was not reflected in the results of colour testing by analytical methods. Interestingly, the colour of jam is one of the most important attributes of acceptance among consumers. The production of blueberry jam is designed to minimise losses of anthocyanins, which significantly affect the colour of the jam [47]. The colour of berry jam is dependent on the content of anthocyanin dyes and on the presence of purple and brown compounds formed during the degradation, polymerisation and condensation of anthocyanins and during non-enzymatic browning reactions [48,49]. Long-term storage at room temperature can cause changes in the colour of jams, which negatively impacts the assessment of these products by consumers [50].

## 5. Conclusions

As a result of this research, it was noticed that the majority of respondents have difficulty distinguishing and properly understanding the terms on the label. It has also been shown that a significant group of respondents misinterpret the “use by” date, thinking that it only applies to changes in food quality. On the other hand, a similar group of respondents consume food after the “best before” date. With regard to durable foods, our analysis showed that the products we tested were safe and free of microbiological contamination on the “best before” date, as well as after 3 and 6 months of storage under the conditions recommended by the manufacturer. This proves that tested groups of food marked as “best before” are safe from the point of view of food microbiology and does not pose a health risk; hence, it can be safely consumed even 6 months after the date of minimum durability.

On the other hand, the length of storage after the “best before” date introduced changes in quality characteristics. The hardness of the pasta decreased, the colour of the milk and the mayonnaise became more yellow and the blueberry jam became browner. The observed changes may affect the perception of the product as spoiled, and—in combination with inadequate knowledge about food labelling—may promote attitudes that can result in food being thrown away by consumers. In order to reduce food waste in households, it would be worthwhile to raise public awareness on food labelling and safety.

In addition, the guarantee of microbiological food safety after the “best before” date, confirmed in our study, opens up the possibility of redistributing these food for social purposes. Based on our research, it is reasonable to indicate a date 1 month after the “best before” date as the minimum (without harm to the quality characteristics and food safety), which would create a chance to properly secure selected food and donate it to charity organisations, for example. However, it should be underlined that these study were conducted only on four selected products and cannot be extrapolated on others foodstuff. In conclusion, further comprehensive durability studies of various food categories should be continued in order to reduce food loss and waste. Also, consumer health and public opinion should be taken into consideration.

## Figures and Tables

**Table 1:** Sociodemographic characteristics of consumer groups (%).

Gender	Women Men	51.1 48.9
Age	18–24 years old 25–34 years old 35–44 years old 45–59 years old over 60 years old	8.3 19.0 18.0 27.4 27.4
Place of Origin	Village City with up to 50,000 residents Cities with 50,001 to 100,000 residents Cities with 100,001 to 200,000 residents Cities over 200,001 up to 500,000 residents Cities over 500,001 residents	38.2 24.8 7.4 9.1 9.0 11.6
Educational Level	Primary school Basic vocational Secondary school Higher	8.4 31.9 42.0 17.7
Subjective Assessment of One’s Financial Situation	Very good Rather good Average Rather bad Very bad No answer	3.6 37.9 53.5 3.5 0.6 0.8

**Table 2:** Details of foodstuffs.

Food Product	Name of Product: Ingredients	Details (Name of Producer; Gross Weight; Type of Packaging Material)	Declared Certifications
Milk	UHT milk, fat content 3.2%	SM Mlekovita, Poland; 500 ml; stored below 25°C, FSC-certified carton from SIG Combibloc	FSSCS 22000, ISO9001, ISO14001
Pasta	Pasta, gluten-free: corn flour, water	Pol-FOODS, Poland; 500g; stored in dry, cool place, plastic bag, 05 PP	-
Mayonnaise	Mayonnaise: refined rapeseed oil, mustard, water, chicken egg yolk	Społem, Poland; 310 ml; glass jar, packed in a protective atmosphere, stored at 2 °C –20°C in a dark place	Q certificate, “JakośćTradycja” Certificate
Jam	Blueberry jam: blueberries (35%), water, sugar, pectin, guar gum, citric acid, sodium citrate	Agros Nova, Poland; 280 g; glass jar, pasteurized	-

Note: Information from labels of product packaging.

**Table 3:** Consumer declarations on consumption after the expiry date (%).

Demographic Variable	Chi-Squared Test	Category	Yes, Regardless of the Type of Food	Yes But Only Durable Products, e.g. Barley/Groats or Pasta	Yes But Only Short-Lived Products, e.g. Yoghurt	No, I Never Consume Products After the Expiry Date	I don’t Know because I don’t Check the Dates	It’s Hard to Say
TOTAL	-	-	7.0	19.5	8.5	48.9	11.2	4.9
Gender	0.000	Woman	7.0	21.2	8.8	52.9	7.1	3.0
		Man	6.9	17.7	8.2	44.8	15.4	6.9
Age	Ns							
Education	0.002	primary school	9.1	19.5	10.6	35.1	22.7	3.1
		basic vocational school	7.8	21.7	7.7	45.5	13.1	4.3
		secondary school	6.4	17.8	9.3	51.2	8.3	7.0
		university/college	5.9	19.6	7.0	55.8	9.5	2.2
Place of Residence	0.000	countryside	8.3	21.1	7.5	44.3	13.2	5.5
		city with up to 50,000 residents	9.5	13.9	12.6	48.0	13.2	2.7
		city with 50,001–100,000 residents	3.8	37.2	6.6	42.3	8.0	2.3
		city with 100,001–200,000 residents	6.4	11.1	13.5	56.4	5.0	7.7
		city with 200,001–500,000 residents	5.3	26.5	0.7	50.4	11.2	6.0
		city with over 500,001 residents	0.8	14.3	6.4	64.9	6.6	6.9
Assessment of one’s Financial Situation	0.000	very good	9.6	4.7	14.3	55.2	6.5	9.7
		rather good	4.2	17.5	7.4	53.9	12.1	4.9
		average	7.9	22.3	9.5	46.1	9.4	4.8
		rather poor	14.6	14.5	2.2	31.5	32.8	4.4
		Poor	39.6	11.2	0.0	49.3	0.0	0.0

ns–not significant.

**Table 4:** Importance of the expiry date of purchased products in the opinion of consumers and knowledge of basic related terms.

No.	Answers	% of Answers	*p*-Value				
			Gender	Age	Education	Place of Residence	Assessment of Financial Situation
The significance of the information found on the label of the purchased product(n = 945)	Expiration Date*	92.1	ns	ns	0.007	0.025	ns
	country or place of origin*	64.3	ns	ns	ns	ns	ns
	name of the company or entity that released the product to the market*	60.9	ns	ns	ns	ns	ns
	nutritional and caloric value*	62.0	ns	0.018	0.001	0.001	ns
	list of ingredients*	70.6	ns	0.017	0.000	0.027	0.036
	Price*	92.4	ns	ns	0.155	0.000	0.003
	storage conditions*	80.8	ns	ns	0.436	0.000	ns
The information on the packaging labelled as “use by” and “best before” mean the same thing (n = 1115)	yes	42.8	ns	ns	ns	0.008	ns
	no	36.0					
	It’s hard to say	21.2					
Products on which the term “best before” is placed, in the respondents’ opinion (n=1115)	barley/groats	40.0	0.007	ns	ns	0.009	ns
	eggs	25.0	ns	ns	ns	0.000	ns
	canned corn	31.8	ns	ns	ns	0.000	ns
	yoghurt, buttermilk, etc.	41.7	ns	ns	ns	0.000	ns
	flour	35.1	ns	ns	ns	0.000	ns
	sandwich meats	32.8	0.020	ns	ns	0.002	ns
	I don’t know	26.8	ns	ns	ns	0.000	ns
The term “best before” means: (n = 1,115)	the date after which the product becomes unsafe for the consumer (may cause poisoning, for example)	39.8	ns	ns	ns	ns	ns
	the date after which the product loses quality (e.g. inferior colour)	37.4	0.005	ns	ns	ns	ns
	the date after which the product cannot be sold	34.5	ns	ns	ns	ns	ns
	the date after which the product can be consumed	9.8	ns	ns	ns	0.004	ns
	It’s hard to say	10.7					

*definitely significant and rather significant answers.ns–not significant.

**Table 5:** The microbiological quality of selected food products on the date of minimum durability (time 0) and 1 month, 3 months and 6 months after the date of minimum durability.

Product	Time [Months]	*Enterobacteriaceae* [log CFU/g]	TVC [log CFU/g]	TYMC [log CFU/g]	*Salmonella* spp.	*Listeria Monocytogenes*
Milk	0	2.03 ± 0.36	2.48 ± 0.35	<1.00	ND	ND
	1	1.15 ± 1.00	1.53 ± 0.40	<1.00	-	-
	3	<1.00	<1.00	<1.00	-	-
	6	<1.00	<1.00	<1.00	ND	ND
Pasta	0	1.77 ± 1.55	1.13 ± 0.98	1.02 ± 0.28	ND	ND
	1	1.51 ± 1.34	2.36 ± 0.37	<1.00	-	-
	3	<1.00	2.29 ± 0.03	<1.00	-	-
	6	<1.00	<1.00	<1.00	ND	ND
Mayonnaise	0	<1.00	1.74 ± 0.04	<1.00	ND	ND
	1	<1.00	1.72 ± 0.36	<1.00	-	-
	3	<1.00	<1.00	<1.00	-	-
	6	<1.00	1.93 ± 0.76	<1.00	ND	ND
Jam	0	1.30 ± 0.52	1.52 ± 0.24	<1.00	ND	ND
	1	2.71 ± 0.30	1.70 ± 0.00	1.32 ± 0.15	-	-
	3	2.32 ± 0.28	2.74 ± 0.45	<1.00	-	-
	6	<1.00	<1.00	<1.00	ND	ND

Explanatory notes: ND–not detected; *- within the columns indicate significant differences within the product categories (*p*< 0.05).

**Table 6:** Physico–chemical analysis of foods tested during storage.

Product	Time [Months]	Water Activity	pH Value
Milk	0	0.99 ± 0.01	6.59 ± 0.09
	1	0.98 ± 0.01	6.59 ± 0.08
	3	1.00 ± 0.01	6.61 ± 0.09
	6	0.98 ± 0.01	6.56 ± 0.08
Pasta	0	0.48 ± 0.02	-
	1	0.49 ± 0.02	-
	3	0.53 ± 0.00	-
	6	0.51 ± 0.01	-
Mayonnaise	0	0.97 ± 0.01	3.80 ± 0.04
	1	1.00 ± 0.01	3.82 ± 0.05
	3	0.96 ± 0.01	3.82 ± 0.05
	6	0.94 ± 0.01	3.7 ± 0.10
Jam	0	1.00 ± 0.04	3.64 ± 0.06
	1	0.96 ± 0.01	3.66 ± 0.04
	3	0.97 ± 0.00	3.67 ± 0.05
	6	0.93 ± 0.01	3.65 ± 0.04

Explanatory notes: *-within the columns indicate statistically significant differences within the product categories (*p*< 0.05).

**Table 7:** Selected texture parameters characterising the food products tested.

Time	Yield Stress of Mayonnaise [N/m2]	Viscosity [N] of Jam	Hardness [N] of Pasta
0	113.80 ± 7.98	0.23 ± 0.05	0.48 ± 0.11
1	106.77 ± 8.07	0.23 ± 0.05	0.32 ± 0.02 *
3	108.87 ± 6.22	0.24 ± 0.03	0.32 ± 0.02 *
6	106.27 ± 2.93	0.23 ± 0.02	0.32 ± 0.02 *

Explanatory notes: *-within the columns indicate statistically significant differences (*p*< 0.05).

**Table 8:** Changes in colour parameters of the tested food products.

	Time	Milk	Pasta	Mayonnaise	Jam
L	0	94.50 ± 0.64	76.17 ± 1.46	90.96 ± 0.92	18.72 ± 0.36
	1	89.25 ± 0.69 *	76.40 ± 1.55	85.68 ± 0.38 *	18.70 ± 0.37
	3	89.50 ± 0.30 *	75.72 ± 1.38	84.15 ± 0.39 *	18.75 ± 0.35
	6	89.14 ± 0.71 *	76.19 ± 1.30	84.90 ± 0.94 *	18.98 ± 0.14
a *	0	−5.07 ± 0.05	−2.83 ± 0.77	−1.55 ± 0.39	10.71 ± 0.75
	1	−3.18 ± 0.04 *	−3.38 ± 1.01	−1.20 ± 0.19	10.68 ± 0.75
	3	−3.20 ± 0.04 *	−1.97 ± 1.24 *	−1.19 ± 0.19	10.53 ± 0.85
	6	−3.22 ± 0.07 *	−2.99 ± 0.97 *	−1.20 ± 0.15	10.05 ± 0.51
b *	0	5.62 ± 0.86	50.87 ± 1.76	18.87 ± 0.58	11.23 ± 0.26
	1	5.93 ± 0.95	52.02 ± 2.29	19.48 ± 0.43	11.24 ± 0.28
	3	6.24 ± 0.41	47.27 ± 4.14 *	19.48 ± 0.30	11.19 ± 0.25
	6	6.22 ± 0.68	50.84 ±3.27 *	19.31 ± 0.36	10.97 ± 0.32

Explanatory notes: L - refers to the luminosity or lightness component, a* - to the intensity of red (+) and green (−) and b* - indicates the intensity of yellow (+) and blue (−); * - within the columns indicate statistically significant differences within the product categories (*p*< 0.05).
